# Highly sensitive ultrasound detection using nanofabricated polymer micro-ring resonators

**DOI:** 10.1186/s40580-023-00378-2

**Published:** 2023-06-20

**Authors:** Youngseop Lee, Hao F. Zhang, Cheng Sun

**Affiliations:** 1 Department of Biomedical Engineering, Northwestern University, Evanston, IL 60208 USA; 2 Department of Mechanical Engineering, Northwestern University, Evanston, IL 60208 USA

**Keywords:** Photoacoustic imaging, Micro-ring resonator, Ultrasound detector, Nano-fabrication, Maskless lithography, Nanoimprint lithography, Soft nanoimprint lithography

## Abstract

Photoacoustic (PA) imaging enables noninvasive volumetric imaging of biological tissues by capturing the endogenous optical absorption contrast. Conventional ultrasound detectors using piezoelectric materials have been widely used for transducing ultrasound signals into the electrical signals for PA imaging reconstruction. However, their inherent limitations in detection bandwidth and sensitivity per unit area have unfortunately constrained the performance of PA imaging. Optical based ultrasound detection methods emerge to offer very promising solutions. In particular, polymer micro-ring resonators (MRRs) in the form of integrated photonic circuits (IPC) enable significant reduction for the sensing area to 80 μm in diameter, while maintaining highly sensitive ultrasound detection with noise equivalent pressure (NEP) of 0.49 Pa and a broad detection frequency range up to 250 MHz. The continued engineering innovation has further transformed MRRs to be transparent to the light and thus, opens up a wide range of applications, including multi-modality optical microscope with isometric resolution, PA endoscope, photoacoustic computed tomography (PACT), and more. This review article summarizes and discusses the evolution of polymer MRR design and the associated nanofabrication process for improving the performance of ultrasound detection. The resulting novel imaging applications will also be reviewed and discussed.

## Introduction

Photoacoustic (PA) imaging enables noninvasive volumetric imaging of biological tissues by capturing the endogenous optical absorption contrast [1–3]. PA employs a short-pulsed laser beam to illuminate the biological tissues. The absorbed laser energy leads to a transient thermo-elastic expansion and subsequently generates ultrasonic pressure waves containing a wide range of frequency components. Detecting the temporal variations of the induced ultrasonic pressure wave allows the construction of the optical absorption contrast-based images. In comparison with the commonly used confocal optical microscopy, PA offers several distinct advantages for imaging biological tissue. Firstly, as the scattering of ultrasonic waves is two orders of magnitude lower than that of the light, it allows PA to image much deeper into the biological tissues [4, 5]. Secondly, since the amplitude of the generated ultrasonic waves are determined by the product of the optical absorption coefficient and the optical fluence, a PA image is better suited for quantifying volumetric optical absorption contrast distribution in tissue. Finally, the depth information can be back calculated from the time-of-flight date buried in the temporal domain of the recorded PA waveforms and thus, a volumetric image in three-dimensions (3D) only requires much faster spatial samplings in two-dimensions (2D). PA can image a wide range of optical absorption contrasts, including endogenous sources, such as hemoglobin and melanin, and exogenous sources, such as chemical dyes, nanoparticles, and reporter gene products. The wide abundance of the available contrast sources makes PA a unique candidate to image abundant anatomical and functional features deep into the biological tissues.

Over the past decades, the evolution of the PA imaging technologies has substantially improved its performance, allowing volumetric imaging deep into the biological tissues at much higher image resolution. In comparison with the early implementation of the acoustic resolution photoacoustic microscope using focused acoustic detector, optical resolution photoacoustic microscope (OR-PAM) further employs a focused incident laser beam to spatially confine the PA generation in improving the lateral resolution. It allows volumetric imaging deeper into the tissue than with confocal microscopy at a given optical irradiation wavelength. However, its attainable imaging depth is still constrained by the optical scattering of the incident focused illumination. Photoacoustic computed tomography (PACT) mitigates this issue by exploiting the diffusive light to illuminate deep into the tissues, delivering ultrasonically defined spatial resolution far deeper than the optical diffusion regime around 1 mm, which is beyond the reach of conventional ballistic optical imaging modalities [2, 6].

However, further improving the image resolution and depth was hampered by the limitations in the commonly used piezoelectric detectors. For example, the axial resolution of OR-PAM was constrained by the finite detection bandwidth with a typical frequency response of piezoelectric detectors no more than tens of megahertz. The lateral resolution in OR-PAM was fundamentally limited by the small numerical aperture (NA) necessitated by the long working distance required to accommodate the bulky and opaque piezoelectric detectors. Optically transparent ultrasound detectors allow the use of high NA objective lens to improve the lateral resolution and integration with other imaging modalities to capitalize on their complementary advantages and minimize their drawbacks. Recently, OR-PAM using transparent piezoelectric ultrasound detectors have been demonstrated by using polyvinylidene fluoride (PVDF) [7], lithium niobate (LN) [8, 9], and a single crystal lead magnesium niobate-lead titanate (PMN-PT) [10]. They overcome the limitation of conventional piezoelectric ultrasound detectors in OR-PAM by simplifying coaxial alignment of optic and acoustic paths and realizing integration with other imaging modalities. However, the transparent piezoelectric ultrasound detectors need further improvements to increase detection sensitivity and frequency bandwidth [11]. PACT further requires a diffraction-limited ultrasound point detector for maximizing the spatial resolution and the field-of-view (FOV). However, due to the limited sensitivity per unit area, the piezoelectric detectors suffer from significantly reduced sensitivity when their detection area is scaled down to a comparable scale of the acoustic wavelength in water.

In contrast, optical-based ultrasound detection offers distinct advantages in tackling the above-mentioned limitations [12, 13]. A variety of optical-based ultrasound detectors being developed have shown promise in greatly improving the detection sensitivity over a wide frequency range. Examples of optical-based ultrasound detector include free space optics-based sensors [14–17], prism-based sensors [18–20], and optical fiber-based sensors [21–25]. By exploiting the strong optical resonance, the integrated photonic devices, including silicon-on-insulator resonators with Bragg gratings [26, 27] and polymer micro-ring resonators (MRRs) [28–31], further reduced the sensing area to be comparable or even smaller than the subjecting acoustic wavelength. Among them, the polymer MRR has been proven to be the most versatile choice. Being optimized over more than a decade by multiple research groups, it provides the high sensitivity with high quality factor (Q-factor) of ~ 10^5^, a broad detection frequency range up to 250 MHz, a small detection area with a size less than 80 μm in diameter, and the highly desirable optical transparency. Collectively, it successfully pushes the normalized detection limit to 1.8 × 10^− 3^ mPa mm^2^/Hz^1/2^, which represents more than one order of magnitude improvement than all other ultrasound detectors, as reported in the recent review article [13]. Its unique capabilities have since enabled a wide range of novel PA imaging systems: its broad detection bandwidth has thus enabled isometric multimodal photoacoustic microscope (PAM) [32], and its optical transparency and miniature form-factor has granted the development of PA endoscope [33] and smart cranial window for longitudinal in vivo PAM imaging [34]. More recently, its miniaturized form-factor and high sensitivity allowed the development of deep-tissue high-frequency 3D PACT with a large FOV [35].

This manuscript aims to provide a comprehensive review on the development of MRR-based ultrasound detectors from the prospective of nano-fabricated integrated photonic devices. We categorize the demonstrated MRR devices based on the associated nanofabrication processes, including maskless lithography, nanoimprint lithography (NIL), and soft nanoimprint lithography (sNIL). The inherent trade-off between process flexibility, scalability, device performance, and their representative applications for PA imaging are compared and analyzed. We hope this review article will provide a practical guideline for researchers to identify the suitable MRR ultrasound detector configurations and the associated nanofabrication process for their PA imaging experiments.

## Working principle of MRR for PA detection

**Fig. 1:**
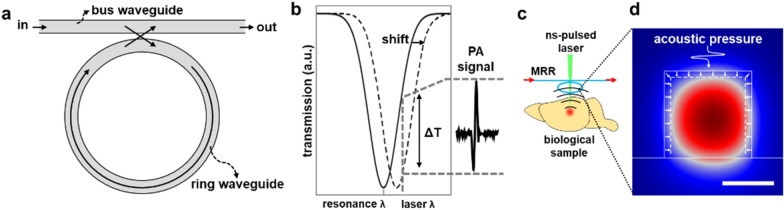
Working principle of MRR-based ultrasound detection. **a** A schematic illustration of the MRR consisting of the ring waveguide and the bus waveguide separated by a low dielectric gap. **b** A representative resonance spectrum of the MRR and intensity-based PA signal detection using a narrow band laser. The shift in the resonance wavelength can be detected as the intensity modulation of the transmissive optical signal at the output port by using the narrow band laser and then converted to the electric PA signal through the photodetector. **c** A schematic illustration of MRR based PA signal generation. An acoustic wave from a biological sample photoacoustically generated by a pulsed laser deforms the polymer MRR resulting in the change of its effective optical path length. This induces the shift of the resonance spectrum of the MRR described in the panel (**b**). **d** A schematic illustration of deformation of the polymer MRR for an incident acoustic pressure with numerically calculated mode profile of ring waveguide. Scale bar 500 nm

Figure 1a illustrates a representative configuration of the MRR that consists of a ring waveguide and a matching straight bus waveguide separated by a low dielectric gap. The bus waveguide serves as the light input and output ports. The light entering the bus waveguide is evanescently coupled into the ring waveguide across the low dielectric gap. The coupled light wave circulates inside the ring waveguide leading to a strong optical resonance via the whispering-gallery-mode [36]. At the resonance wavelength, the light circulating within the ring waveguide coupling back to the bus waveguide forms a deconstructive interference, which results in a dip in the transmission spectrum (Fig. 1b). In PA imaging, the photoacoustically generated ultrasonic pressure waves impinging upon the MRR waveguide will cause it to deform (Fig. 1c). The changes in its cross-sectional area and the refractive index due to the elasto-optic effect alter its effective optical path length within the ring waveguide, and subsequently results in a shift in its resonance wavelengths (Fig. 1d). Thus, one would expect the resonance wavelength shift to be proportional to the mechanical compliance of the ring waveguide. Using a narrow band laser source to interrogate the MRR, the shift in the resonance wavelength can be conveniently transduced into the intensity modulation of the transmitted signal at the output port [37], and subsequently converted into electric PA signal using a photodiode detector (Fig. 1b). The modulation of the PA signal is proportional to the slope of the resonance curve, which is determined by the Q-factor of the MRR. Thus, MRRs featuring higher Q-factors are also of the interests for increasing the ultrasound detection sensitivity.

The sensitivity of detecting ultrasonic pressure through the MRR can be estimated by the following Eqs. [30, 37]:

1 $$S=\frac{dT}{dP}=\frac{d{\text{n}}_{\text{e}\text{f}\text{f}}}{dP}\frac{d{\lambda }_{R}}{d{\text{n}}_{\text{e}\text{f}\text{f}}}\frac{dT}{d{\lambda }_{R}},$$

where *T* is the transmission at the output port through the bus waveguide, *P* is the ultrasonic pressure, n_eff_ is the effective refractive index, and *λ*_*R*_ is the resonance wavelength of the MRR. The resonance wavelength of the MRR with the ring radius *R* is defined as [38]:

2 $${\lambda }_{R}=\frac{2\pi R{\text{n}}_{\text{e}\text{f}\text{f}}}{\text{m}}, \quad \text{m}=1, 2, 3 \ldots$$

The first term of the Eq. (1) $d{\text{n}}_{\text{e}\text{f}\text{f}}/dP$ is the pressure-induced effective refractive index change of the MRR considering Young’s modulus and elasto-optic coefficient of the waveguide materials, and cross-sectional deformation of the ring waveguide. The second term $d{\lambda }_{R}/d{\text{n}}_{\text{e}\text{f}\text{f}}$ defines resonance wavelength shifts due to changes of the effective refractive index, which can be estimated to ${\lambda }_{R}/{\text{n}}_{\text{e}\text{f}\text{f}}$for a small perturbation in n_eff_. The third term $dT/d{\lambda }_{R}$ can be defined as the slope of the resonance curve, which can be approximated as linearly proportional to the Q-factor of the MRR. Therefore, the use of soft materials to construct the MRR, and a longer resonance wavelength and a higher Q-factor of the MRR can favorably increase the detection sensitivity for ultrasonic pressure.

The angular dependent ultrasound detection characteristic of the polymer MRR has been studied systematically by Zhang et al. [39]. The ultrasound detection using the ring waveguide was mathematically expressed as the Rayleigh integral, which is equivalent to the pressure generated by a ring-shaped piston. An analytic model was first developed to study the steady-state response to the continuous ultrasonic waves, while a numerical model was developed to analyze the transient response to the photoacoustic-induced impulsive waves. Both analytic and numerical models are validated by experimental studies. The angular ultrasound detection sensitivity is determined by the dimension of MRR with respect to the ultrasound wavelength. Thus, MRRs in a miniaturized form-factor, with the diameter of tens of micrometers, offer distinct advantage for wider angular detection range in comparing with the conventional area detectors. This study established a theoretical framework for quantitatively analyzing the trade-off between the FOV, ultrasound detection sensitivity, detection bandwidth, and axial resolution, which can be used to optimize the PAM for various applications in biomedical imaging and diagnostics.

## Nanofabrication methods

Designing the MRR to optimize its sensitivity for detecting the ultrasound signals relies on mitigating the delicate balance between its mechanical compliance and the Q-factor. As an example, optical resonators made of silicon dioxide (SiO_2_) can provide extremely high Q-factors up to 10^10^ [36], but the use of rigid materials compromises its ability to effectively transduce the incident ultrasonic pressure wave into the measurable quantity of the resonance wavelength shifts. On the other hand, fabricating the MRR using softer polymeric materials offers many promising solutions. However, the selection of the materials and the matching fabrication process need to be further optimized to maximizing its Q-factors. Notably, the Q-factor of the MRR is ultimately determined by the combination of the coupling loss between the ring waveguide and the bus waveguide, as well as the intrinsic loss when light is circulating within the ring waveguide. The intrinsic loss further comprises the materials absorption loss and the scattering loss within the ring waveguide. The optimal Q-factor of the MRR can be obtained at the critical coupling conditions, at which the coupling loss matches the intrinsic loss [38].

The chase for higher sensitivity has thus imposed stringent requirements in the design and fabrication of MRR for optimal performance in ultrasound detection. Specifically, it requires (1) the MRR to be fabricated using soft polymeric materials with low optical absorption loss; (2) the ring waveguide feature deep sub-wavelength surface smoothness to minimize the scattering loss, (3) that the low dielectric gap between the ring waveguide and the bus waveguide can be reliably fabricated to reach the critical coupling condition, and (4) a miniaturized form-factor so MRR can be approximated as a point detector. Here, we review the developments of MRR-base ultrasound detectors from the prospective of the nano-fabrication processes, including maskless nanolithography, NIL, and sNIL. The trade-off between process flexibility, accessibility, and the performance of the resulting MRR-based ultrasound detector have been thoroughly analyzed. We also discussed the associated applications to highlight the characteristics of the MRR-based ultrasound detectors.

### Maskless nanolithography

**Fig. 2:**
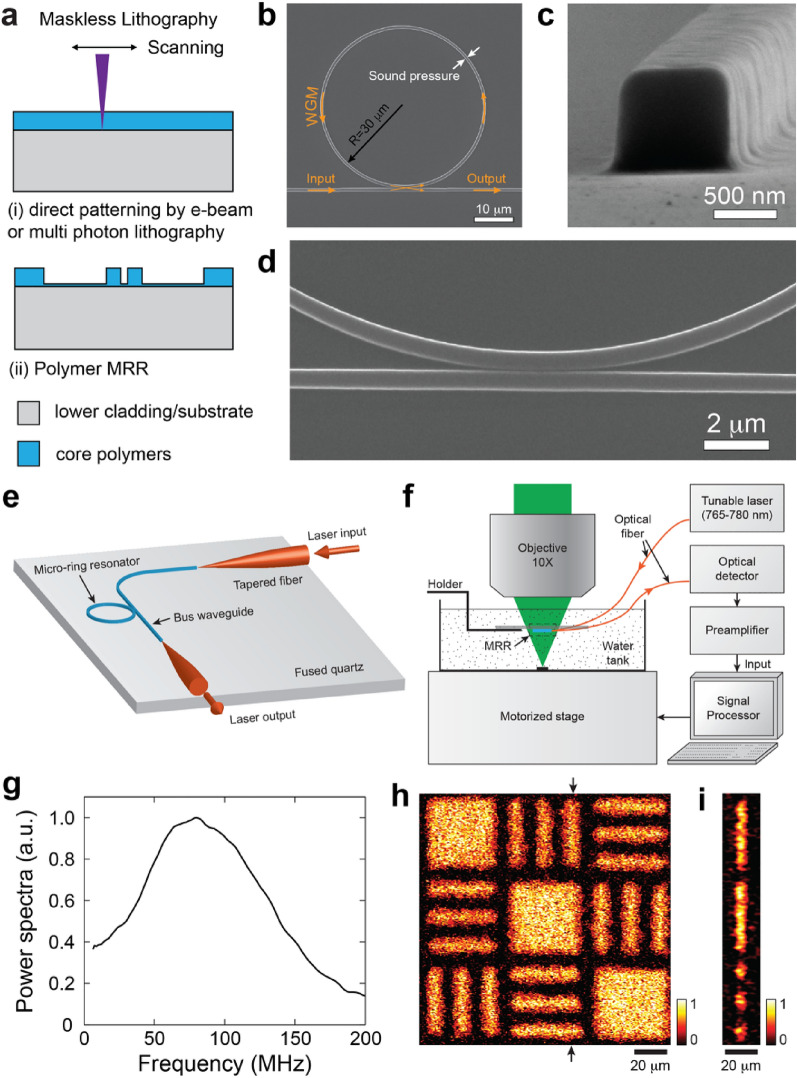
Nanofabrication of polymer MRRs by maskless lithography. **a** A schematic illustration of fabrication procedures of maskless lithography such as EBL and MPL. **b** A top view SEM image of the SU-8 MRR by EBL. **c** A cross-sectional SEM image of the waveguide with a width of 800 nm and a height of 800 nm. **d** Close-up view of the gap of 150 nm between the ring and bus waveguides. **e** A schematic illustration of the packaged SU-8 MRR ultrasound detector coupled to two tapered optical fibers. **f** A schematic illustration of PAM setup with the SU-8 MRR ultrasound detector. **g** A spectral profile of the impulse response for the MRR based PAM system after a 10-point moving averaging. The 3-dB frequency bandwidth is 140 MHz. **h** The MAP image of the target along the x–y plane from the reconstructed volumetric image. **i** A cross-sectional image of the target at the position indicated by the arrows in the panel (**h**) (Reproduced with permission from [30])

In the conventional maskless nanolithography processes, patterns are generated via scanning a focused beam via either a raster or vector fashion digitally, without the need for a set of photomasks. It enhances the process flexibility, but at the burden of high unit cost due to its series patterning nature, and as such is best suited for fabricating devices requiring frequent design iterations with quick turnaround. Ultraviolet (UV) projection based maskless pattern tools do offer increased patterning speeds, but they are not readily available for creating nanometer scale features due to the diffraction limited resolution. The most commonly used maskless nanolithography process for fabricating polymer MRRs are electron beam lithography (EBL) [40, 41] and multi photon lithography (MPL) [42, 43] (Fig. 2a).

EBL has been widely used for patterning polymeric materials at nanometer resolution. Li et al. used EBL to fabricate the SU-8 MRR on a 250-µm-thick quartz substrate. The MRR has a diameter of 60 μm, a cross-sectional area of 800 × 800 nm^2^, and a 150 nm-wide air gap in-between the ring waveguide and the bus waveguide (Fig. 2b–d) [30]. The in- and out-coupling of the bus waveguide were accomplished using tapered optical fibers (Fig. 2e). The highest Q-factor of 1.0 × 10^4^ has been demonstrated at the driving wavelength near 780 nm. Figure 2f illustrates the PAM system using the SU-8 MRR ultrasound detector. Test samples were placed at the bottom of a water tank used for an acoustic coupling medium and are mounted on a 2D translational stage. The MRR ultrasound detector was immersed in water and a nanosecond pulsed laser (*Elforlight Ltd.*) at 532 nm as the PA excitation laser was focused onto the samples by a 10× objective lens with NA of 0.25 through the optically transparent MRR ultrasound detector. The optical axis of the PA excitation laser was carefully aligned with the center of the MRR. They used a narrow band tunable laser (TLB-6712, *New Focus*) with a tunable wavelength range of 765 to 780 nm as an interrogating laser source for the MRR. Transmitted light signal through the MRR was collected by an avalanche photodetector (APD) (APD210, *Menlo Systems*) and digitized after being amplified by 28 dB. The optical ultrasound detector of the SU-8 MRR achieved the NEP of 6.8 Pa over a broad frequency bandwidth from DC to 140 MHz (Fig. 2g). Fabricating the polymer MRR on the transparent quartz substrate facilitates easy integration of the optical ultrasound detector co-axial with the optical axis of the PA excitation beam, thereby allowing the utilization of high-NA objective lenses for improving the lateral resolution [30]. Finally, a lateral resolution of 2.0 μm and axial resolution of 5.3 μm in PA imaging had been demonstrated using a standard resolution target made of carbon-black materials (Fig. 2h and i). It is worthwhile to note that, being commonly recognized as a 2D patterning tool, EBL is better suited for fabricating low-aspect-ratio patterns due to the omnidirectional backscattering of electrons. Thus, the process has rather limited yield in reliably fabricating the low dielectric gap that is 150 nm wide and 800 nm high.

**Fig. 3:**
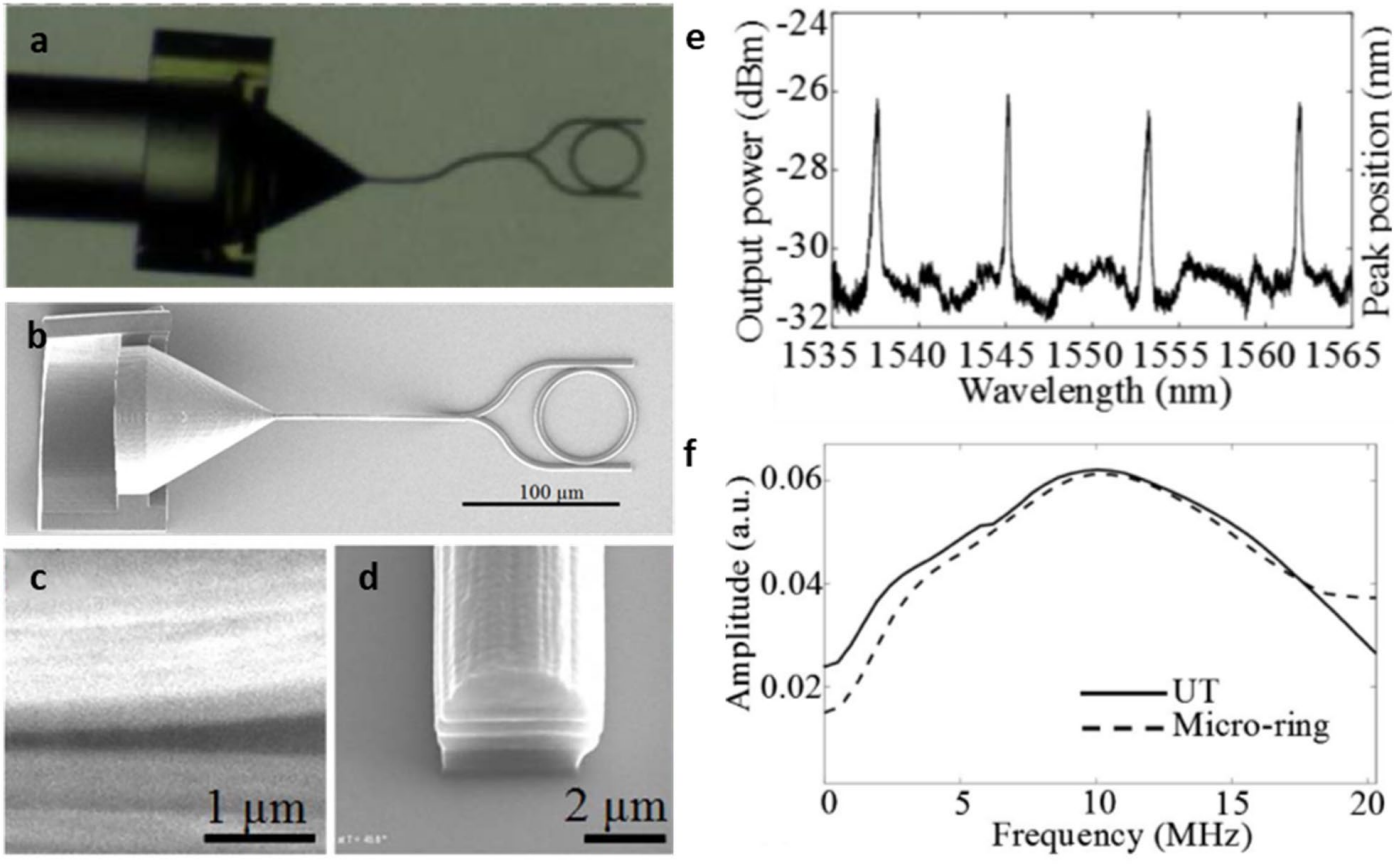
**a** An image of the integrated polymer MRR with the SMF obtained by a CCD camera. **b** A top view SEM image of the MRR with IP-Dip polymer fabricated by MPL. **c** Close-up view of the gap between the ring and the straight waveguide. **d** High-magnification view of the waveguide cross-section with a diameter of 4 μm. **e** The resonance spectrum of the polymer MRR. **f** Spectral response profiles of ultrasound transducer (UT) and the MRR (The reproduced with permission from [46])

Alternatively, MPL offers a solution for fabricating 2D and 3D photonic structures with sub-100 nm resolution [44], sufficient for fabricating polymer MRRs operating at the visible and near infrared (NIR) spectral range. Melissinaki et al. used MPL to directly pattern a MRR made of inorganic-organic hybrid composite onto the tapered region optical fibers (OFT), with the demonstrated Q-factor 2.6 × 10^3^ at 1.55 μm [45]. The direct fabrication of MRR on the OFT eases the burden for the system integration, but at the cost of compromising its ability to fine tune the in- and out-coupling of the MRR. More recently, Wei and Krishnaswamy successfully demonstrated an optical ultrasound detector with a polymer MRR fabricated using a commercial multiphoton 3D lithography tool (Photonic Professional GT2, *Nanoscribe*) [46]. The ability to fabricate nanostructures in 3D could alleviate the limitation in patterning the high aspect ratio for the commonly used 2D patterning methods. Furthermore, directly writing a 3D taper coupler can effectively increase the coupling efficiency by accommodating the mismatch in the mode diameter of a single mode fiber (SMF) and the hard cladded waveguides (Fig. 3a). Fabrication of the polymer MRR with a diameter of 60 μm, and a circular cross-section with the radius of 2 μm has been demonstrated (Fig. 3b–d). After development, the MRR fabricated using the commercial photoresist (IP-Dip) had demonstrated an improved Q-factor of 3.4 × 10^3^ at 1.55 μm and can detect ultrasound signal from the ultrasonic transducer over a bandwidth of 10 MHz at a detection sensitivity of 289.16 mV/MPa (Fig. 3e and f). Despite the inherent process flexibility, MPL also suffers from some constraints. The selection of the materials is limited to the available photoresists compatible with the MPL process. Besides, the high capital cost of the MPL makes its accessibility rather limited. EBLs are considerably more expensive but they are more accessible in many of the shared cleanroom facilities.

**Fig. 4:**
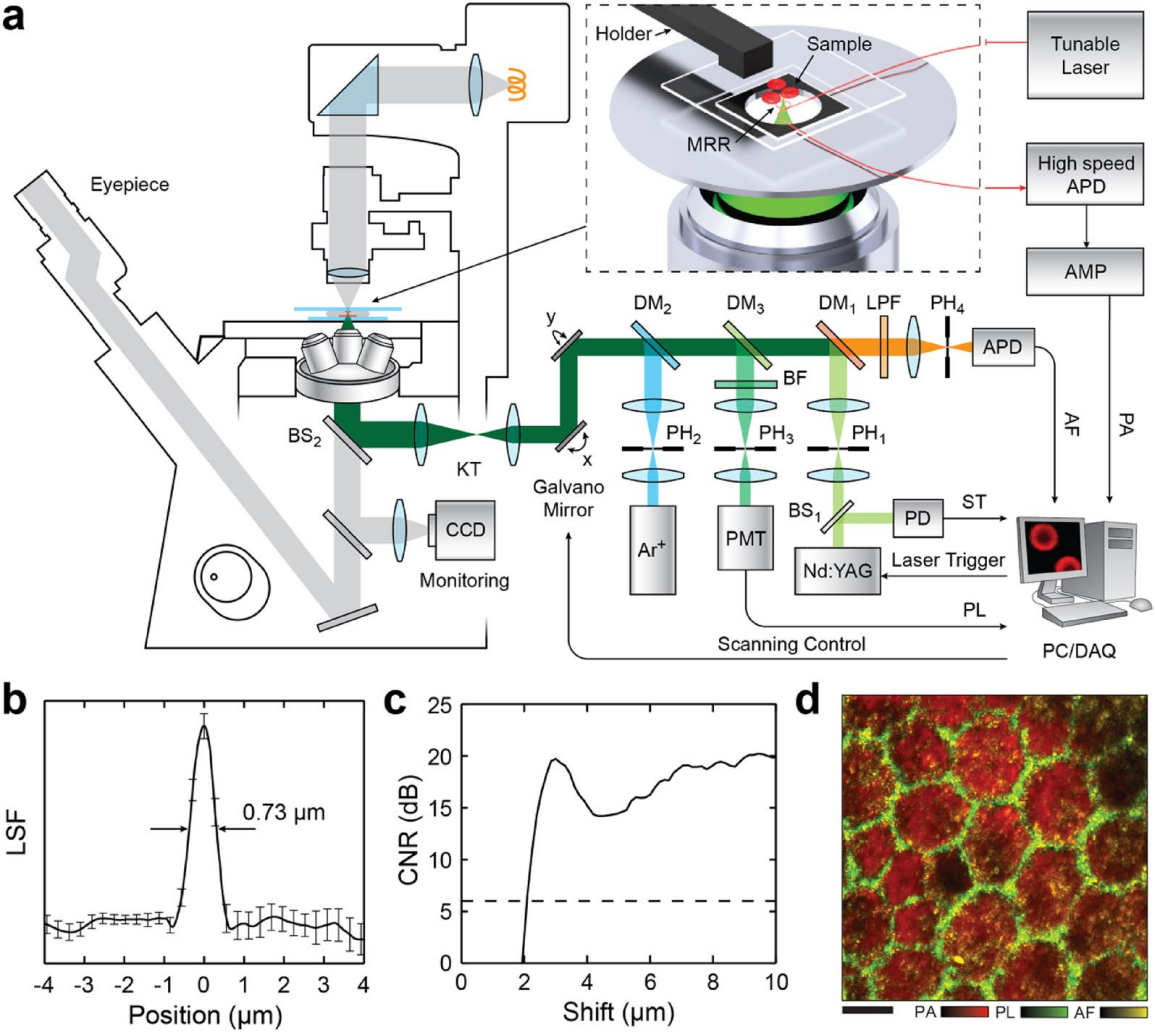
**a** A schematic illustration of the multimodality microscopy system combining laser-scanning confocal microscopy and PAM with the optically transparent polymer MRR. **b** Line spread function (LSF) calculated by the gradient of the edge spread function of the carbon black thin film target. The lateral resolution of 0.73 μm is determined by the full width at half maximum of the LSF. **c** Contrast-to-noise ratio (CNR) profile. Estimated axial resolution of 2.12 μm by a shift-and-sum method. **d** An overlaid image of the flat mount RPE tissue sample from three image modalities: MAP of PAM image, actin-stained fluorescent confocal image, and autofluorescence confocal image of lipofuscin. Three modalities acquired simultaneously. Scale bar 10 μm (Reproduced with permission from [32])

Nevertheless, the nature of the maskless nanolithography process does offer the flexibility for researchers quickly engaging in the implementation of a MRR detector for PA imaging applications and has thus enabled some novel PA imaging platforms. Fabricating a polymer MRR on a 250-µm-thick quartz substrate made the MRR ultrasound detector fully transparent to the light and thus, allows the ultrasound detector to be placed along the primary optical axis of PA excitation. As a result, Dong et al. demonstrated the ability to transform a commercial optical microscope into a multi-modality microscope by simply inserting the transparent MRR ultrasound detector in-between the objective lens and the sample (Fig. 4a) [32]. They also integrated the optically transparent polymer MRR ultrasound detector into a commercial inverted optical microscope platform to achieve further improvements in the axial resolution, which is limited due to constraints in the ultrasound detection bandwidth. The thin form-factor of the MRR ultrasound detector can be used with the high-NA objective lens, enabled improved lateral resolution of 0.73 μm (Fig. 4b). The MRR ultrasound detector used in this study exhibited detection frequency bandwidth from DC to 250 MHz, which resulted in a significantly improved axial resolution of 2.12 μm (Fig. 4c). Finally, the multi-modality imaging capability was demonstrated using a flat mount retinal pigment epithelium (RPE) sample. Figure 4d shows the overlaid image of the RPE from three image modalities: maximum amplitude projection (MAP) of PAM image, actin-stained fluorescent confocal image, and autofluorescence confocal image of lipofuscin.

**Fig. 5:**
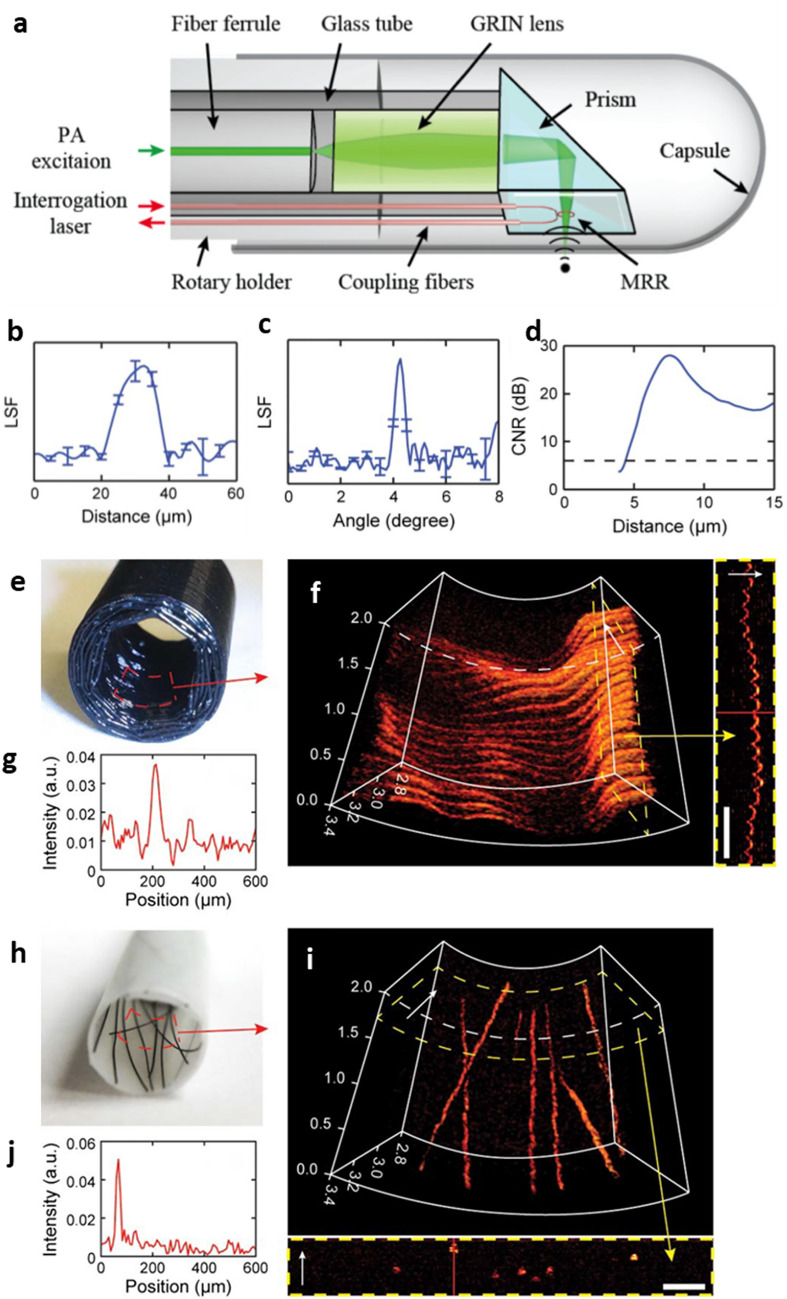
The polymer MRR ultrasound detector for a PA endoscopic probe. **a** A schematic illustration of the PA endoscopic probe. **b** Axial LSF profile. **c** Tangential LSF profile. **d** CNR calculated by the shift and sum method. Radial resolution is estimated at 6 dB (dashed line). **e** Photograph of the black plastic tube phantom. **f** 3D volumetric rendering of PA image of its inner surface. Axial cross-section within the region marked by the dashed box is shown in the right panel. Scale bar: 500 μm. **g** A-line signal from position marked by the red line in panel (**f**). **h** Photograph of the hair phantom sample. **i** 3D volumetric rendering of PA image of the hair phantom sample. The flattened cross-sectional view within dashed region is shown in the lower panel. **j** A-line signal from the position marked by the red line in panel (**i**). Scale bar 500 μm (Reproduced with permission from [33])

The optical transparency and the miniaturized form-factor of the MRR ultrasound detector further enabled the demonstration of a PA endoscopic probe [33]. The MRR ultrasound detector was integrated into a compact PA endoscopic probe with an outer diameter of 4.5 mm (Fig. 5a). The probe consists of a gradient index (GRIN) lens to focus the PA illuminating from a SMF, a right-angle-prism for side illumination, and the MRR ultrasound detector being directly attached to the side-facet of the prism. The MRR ultrasound detector has the NEP 352 Pa and the frequency bandwidth from DC to 250 MHz. The demonstrated tangential, radial, and axial resolution of the PA endoscopic probe is 15.7 μm, 4.5 μm, and 16.0 μm, respectively (Fig. 5b–d). Volumetric imaging using the PA endoscopic probe was further demonstrated using two phantom samples: a hollow black plastic tube and strands of human hairs (Fig. 5e and h), that mimic actual anatomical features for endoscopic imaging. Ridge-like features with a depth of less than 100 μm are clearly distinguishable in the reconstructed 3D PA rendering (Fig. 5f and g). Randomly distributed strands of human hairs with a diameter of ~ 100 μm are clearly imaged with a good signal-to-noise ratio (SNR) in the reconstructed 3D rendering (Fig. 5i and j).

### Nanoimprint lithography (NIL)

**Fig. 6:**
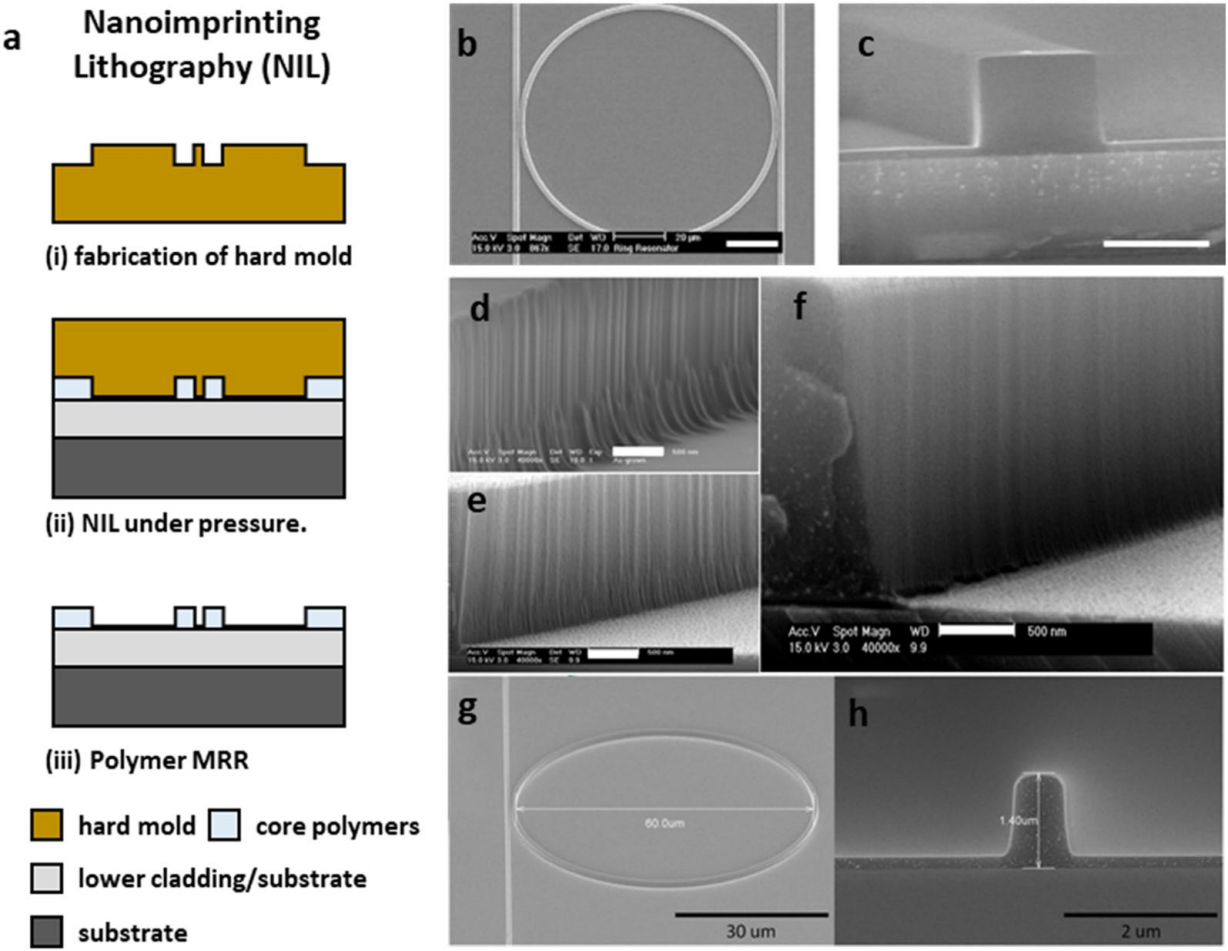
Nanofabrication of polymer MRRs by NIL. **a** A schematic illustration of fabrication procedures of NIL. **b **and **c** Top view and cross-sectional view of SEM images for fabrication results of the PS MRR by NIL with the engraved SiO_2_ pattern on Si mold. Scale bar: **b** 20 μm, **c** 2 μm. Reproduced with permission from [51]. SEM images of PS MRR sidewalls by NIL using the Si mold fabricated by EBL and RIE processes: **d** without PMMA resist reflow process, **e** with PMMA resist reflow process, and **f** with PMMA resist reflow and thermal oxidation process. Scale bar: **d**,** e**,** f** 500 nm, Reproduced with permission from [28]. **g** A SEM image of PS MRR with a diameter of 60 μm fabricated by NIL with the Si mold. Scale bar: 30 μm. **h** A cross-sectional SEM image of the waveguide with a width of 1 μm and a height of 1.4 μm. Scale bar: 2 μm (Reproduced with permission from [60])

While maskless nanolithography processes offer the flexibility to accommodate rapid design changes, they suffer from the high unit cost for manufacturing MRR detectors. In contrast, NIL processes were originally developed as a scaled-up nano-manufacturing tool with intend high throughput and yield. NIL creates a thickness contrast in a polymer layer by pressing a hard mold with surface-relief nanoscale structures into the polymer layer on a substrate under elevated pressure and temperature (Fig. 6a) [47]. In fact, Prof. O’Donnell and Prof. Guo spearheaded the development of polymer MRR-based ultrasound detectors when they both were at the University of Michigan. Their pioneer work published in 2002 reported the first polymer MRR with the Q-factor of 5.8 × 10^3^ operating at 1.55 μm, being fabricated using the NIL process [48]. The polymer MRR with a waveguide height of 1.5 μm was fabricated by combination of EBL, NIL, and reactive ion etching (RIE) processes. The MRR pattern was first created in photoresist layer on a silicon (Si) substrate using EBL. Then, the pattern was transferred to the metal mask by deposition of a titanium (Ti)/nickel (Ni) layer and lift-off process. Using the Ti/Ni layer as the etching mask, the MRR pattern was finally transferred into the underlying SiO_2_ layer to create the hard NIL mold after the removal of the Ti/Ni layer. Using it as the mold, the MRR pattern was then imprinted into a PMMA layer spin-coated on a Si substrate with a thermally grown SiO_2_ cladding layer. The residual PMMA layer was removed by RIE to ensure the fidelity of the MRR pattern transfer process. The initial thickness of the polymer layer was chosen to be 200 nm to minimize the thickness of the residual polymer layer on compressed regions. The NIL process was accomplished at high pressure of 75 kg/cm^2^ to process temperature of 175 °C to assist the polymer flow for effective mold filling.

After the first demonstration of polymer MRR fabrication by NIL, Guo and colleagues have made significant progress in ultrasound detection and PA imaging. They successfully demonstrated polymer MRR-based ultrasound detectors by NIL [37, 49, 50]. The MRRs showed the Q-factor up to 6 × 10^3^ at a working wavelength of 1.55 μm, experimentally demonstrating ultrasound detection with a NEP of 230 Pa and frequency bandwidth of 90 MHz [50]. Demonstrations of capability of the polymer MRR as the ultrasound detector led to the first demonstration of PA tomography (PAT) imaging using the polymer MRR as the optical ultrasound detector [51, 52]. In this work, the MRR made of polystyrene (PS) has a diameter of 100 μm and cross-sectional area of 2 × 2 µm^2^ on the Si substrate with thermally grown SiO_2_ layer. The optical ultrasound detectors with the PS MRRs (Fig. 6b and c) with the Q-factor of 5 × 10^3^ and 6 × 10^3^ showed a NEP of 4.1 kPa with a frequency bandwidth of 70 MHz [51] and a NEP of 111 Pa with a frequency bandwidth of 16 MHz [52], respectively. They achieved a lateral and axial resolution of 150 μm and 90 μm for the Q-factor of 5 × 10^3^ and 209 μm and 81 μm for the Q-factor of 5 × 10^3^ in PA imaging with phantom samples of PS beads. They demonstrated fabrication of the PS MRR on a quartz substrate by NIL for a PA endoscopic probe [53]. The PA endoscopic probe consists of the PS MRR on the quartz substrate, a right-angle prism, and an optical fiber with a core diameter of 550 μm for coupling external PA excitation laser and its outer diameter is ~ 5 mm. They achieved PA imaging of a carbon fiber phantom with the transverse and radial resolution of 750 μm and 21 μm by using the MRR based PA endoscopic probe.

Since the initial demonstrations, efforts have then focused on further optimizing the NIL to improve the Q-factor of the MRR via loss reduction. Ultrasound detectors with higher detection sensitivity allow deeper PA imaging. As shown in Fig. 6d, the mold fabricated by EBL and RIE processes suffers from sidewalls with insufficient roughness to achieve high Q-factors [28, 54]. Sidewall roughness was subsequently transferred into the polymer waveguide, which resulted in the elevated scattering loss of the MRR. To mitigate this issue, additional processes had been reported to smooth the sidewall of the mold being used. Armani et al. demonstrated a microcavity on a chip with ultra-high Q-factor of 1.25 × 10^8^ by using selective reflow process after photolithography and dry etching [55]. They first fabricated the Si micro-disk structure by using photolithography for photoresist patterning, SiO_2_ etching in hydrofluoric acid (HF) solution, and xenon difluoride (XeF_2_) isotropic etching of Si. Then, they made the Si microcavity with smooth sidewalls by using selective reflow with CO_2_ laser at a wavelength of 10.6 μm. The diameter was shrunk from 160 μm to 120 μm after the selective reflow process. The Q-factor was improved from 10^5^ order in the Si micro-disk to 1.25 × 10^8^ at 1.55 μm in the Si microtoroid. Kim et al. reported fabrication of a polymer MRR by NIL using a quartz mold with a silicon nitride (SiN) smoothing buffer layer [56]. They first fabricated the MRR pattern on the quartz substrate by using photolithography, selective etching, and RIE. The 0.4-µm-thick SiN layer was deposited on the quartz substrate by using the low-pressure chemical vapor deposition (LPCVD). The gap between the ring waveguide and the bus waveguide was reduced from 1 μm to 0.2 μm after the deposition of SiN. The additional SiN buffer layer improved the surface smoothness of the mold from the standard deviation from 62.2 nm to 2.2 nm, which is corresponding to estimated scattering loss from 38 dB/mm to 5 × 10^− 4^ dB/mm. They finally demonstrated the polymer MRR with the Q-factor of 1.0 × 10^5^ at 1.55 μm by using NIL of MRR patterns into a lower cladding layer with an UV curable polymer (ZPU445, *ChemOptics*) and spin-coating of a core material with Ormocer polymer. The final optimized process flow was reported by Ling et al. in 2011, with significantly improved Q-factor of 1.5 × 10^5^ at 1.55 μm by using a Si hard mold with smooth sidewalls [28]. As a result, the improved ultrasound detection sensitivity was demonstrated with NEP of 88 Pa and the frequency bandwidth of 74 MHz. They fabricated the Si hard mold by using EBL of PMMA, RIE of SiO_2_ mask with the PMMA mask, and deep Si etching with the SiO_2_ mask. First, they created the MRR pattern on an 800-nm-thick PMMA layer on a 400-nm-thick thermal SiO_2_ layer on a Si wafer. The pattern was transferred to the SiO_2_ layer by RIE with the PMMA as the etch mask after thermal reflow of the PMMA resist and then the PMMA resist was removed in hot acetone. The MRR pattern was transferred onto the Si substrate by deep Si etching with the SiO_2_ etch mask. Finally, the Si surface was further smoothened by thermal oxidation and removal of SiO_2_ layer. They used two important fabrication steps, thermal reflow and thermal oxidation, to smooth the sidewall of the Si mold. They applied thermal reflow of the PMMA resist before the SiO_2_ etch step. The reflowing temperature and time duration were 115 °C for 90 s, which can reflow the PMMA without causing deformation in the gap region. The reflow process can reduce imperfections in the PMMA patterns and harden the edge of PMMA. The sidewall of the PS MRR fabricated by NIL using the Si mold with the PMMA reflow process has relatively small vertical roughness compared to the that of the PS MRR from the Si mold without the reflow process (Fig. 6e). They further smoothed the surface of Si sidewall by thermal oxidation followed by wet etching of a thin SiO_2_ layer after the Si etch step (Fig. 6f). To reduce the propagation loss, Guo and colleagues made a strategic move to shift the operating wavelength from 1.55 μm to 780 nm, to reduce the light absorption in water and PS. Further combining with the reduction of the ring waveguide diameter form 100 μm to 40 μm (Fig. 6g, h), improvement in the Q-factor up to 4.0 × 10^5^ had been successfully demonstrated [29].

**Fig. 7:**
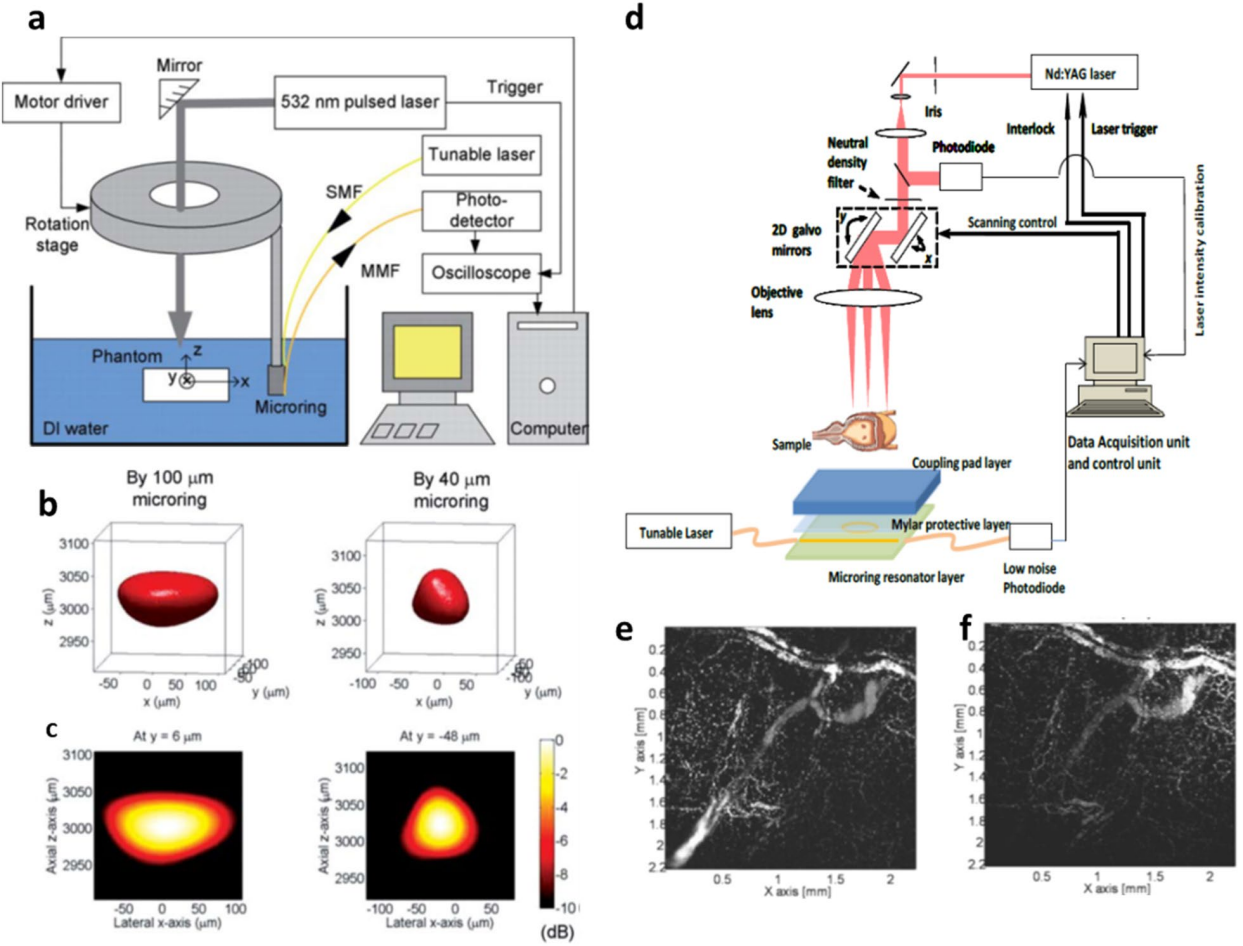
**a** A schematic illustration of PAT setup with the polymer MRR. **b** 3D PA images (-6 dB iso-surface) of a 50-µm PS bead by using polymer MRRs with a diameter of 100 μm and 40 μm. **c** 2D colormap images (10-dB dynamic range) on the plane crossing the central position of the bead. Reproduced with permission from [57]. **d** A schematic illustration of PAM setup with the polymer MRR. **e **and** f** MAP PA images on x-y plane of the ex vivo images of the vasculature in a mouse bladder wall by using the polymer MRR ultrasound detector and conventional piezoelectric transducer (HNC-1500, *Onda*) (Reproduced with permission from [58])

PAT imaging has thus been demonstrated on a phantom sample using the packaged MRR with a SMF and a multimode fiber (MMF) as the input and output ports and a rotational stage (Fig. 7a). The phantom sample and the packaged MRR are placed in the deionized water medium for acoustic coupling during the imaging process. The lateral and axial resolutions are 146 μm and 50 μm for the MRR diameter of 100 μm and 55 μm and 50 μm for the MRR diameter of 40 μm (Fig. 7b and c) [57] and the NEP is 100 Pa and 21.4 Pa over 1 MHz to 75 MHz for a MRR diameter of 60 μm and 40 μm, respectively [29]. The polymer MRR ultrasound detector enabled the realization of an all-optical PAM imaging system with an improved axial resolution, which was constrained by the finite detection bandwidth of conventional piezoelectric ultrasound detectors. They also demonstrated an all-optical PAM system by using PS MRRs on the SiO_2_ deposited Si substrate [58–61]. The PAM systems include the PS MRR ultrasound detector, acoustic coupling medium or layer, samples, a pulsed laser for PA generation, an excitation laser for driving the MRR, a photodiode detector, and data acquisition and control units (Fig. 7d). Scanning of the pulsed laser can be controlled by using a 2D galvanometer mirror [58] or a microelectromechanical system (MEMS) mirror [59]. They demonstrated PAM imaging with the lateral and axial resolution of 5 μm and 8 μm and its NEP of 29 Pa over 1 MHz to 75 MHz by using the PS MRR with the diameter of 60 μm and the Q-factor of 3.0 × 10^5^ at 780 nm and the 2D galvanometer mirror. PA images of the vasculature in a mouse bladder wall acquired by PAM with the MRR ultrasound detector showed comparable results of PAM using the commercial transducer with a high sensitivity providing the NEP of 19 Pa (Fig. 7e and f). They achieved MEMS based PAM imaging with the lateral and axial resolution of 17.5 μm and 20 μm. The axial resolution of their PAM with the polymer MRR was further improved to 2.7 μm [60].

Clearly, the extensive literature reported by Guo and colleagues had substantially improved the performance of polymer MRR-based ultrasound detectors, using a potentially highly scalable NIL process. In their more recent work, they have set the record for Q-factor of 8.0 × 10^5^ for the polymer MRR designated for bio-sensing application [62]. However, the NIL process used was facing its own limitations. Firstly, the fidelity of the NIL process relies on the conformality of the rigid mold and substrate being used, which is constrained by the surface smoothness and curvature of both. Even for the lithographic grade Si wafers with highly polished surface, the surface curvatures still exist. Thus, the NIL process employed large hydraulic pressure to physically deform the rigid mold and substrate, to ensure their proper contact during the imprinting process. The resulting NIL systems are not readily accessible to the general research community. Furthermore, the required hard engagement among two rigid surfaces makes the process vulnerable to the presence of surface defects or asperities.

### Soft nanoimprint lithography (sNIL)

**Fig. 8:**
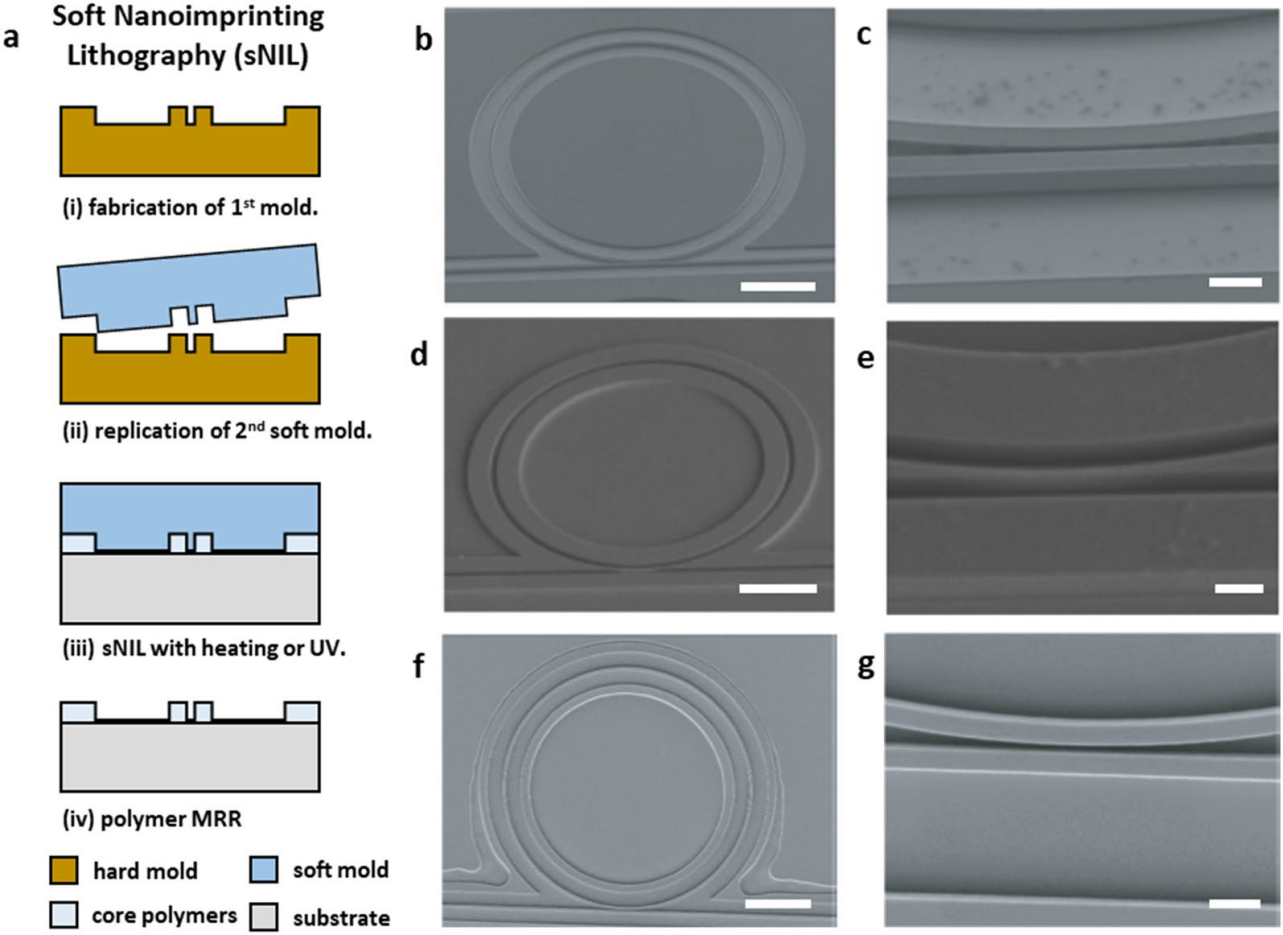
Nanofabrication of polymer MRRs by sNIL. **a** A schematic illustration of fabrication procedures of sNIL. SEM images of the Si hard mold (**b**), the PDMS soft mold (**d**), and the PS MRR (**f**) by sNIL. Scale bar 20 μm. Their corresponding magnified SEM images for the coupling region are shown as (**c**), (**e**), and (**g**). Scale bar 2 μm (Reproduced with permission from [34])

The issues associated with hard contact in NIL process can be overcome by sNIL process using the molds made of soft polymers with low Young’s modulus such as polydimethylsiloxane (PDMS) and perfluoropolyether (PFPE). Fabrication of polymer nanostructures by sNIL follows the fabrication process as shown in Fig. 8a. The initial master template is prepared by using EBL. The soft mold is then replicated from this master template. The soft mold is placed on a polymer coated substrate. The pattern structure of the soft mold is filled with the polymer by capillary force. In this step, additional heating to reduce the polymer viscosity can help the polymer fill the mold pattern perfectly. Then, the soft mold is demolded from the substrate. Since the soft mold can be easily replicated from one expensive master template, cost of fabrication process is significantly reduced [63]. Their flexibility enables intimate and conformal contact between the mold and the substrate without applying high pressure during pattern transfer. In addition, sNIL process is robust against particle contaminants because the soft mold can be locally deformed around particles preventing damages to the mold and the substrate, which leads to a high fabrication yield.

Several demonstrations in the fabrication of MRR with various polymer materials were reported by using sNIL with PDMS [64, 65] and PFPE molds [66–68]. Poon et al. first reported fabrication of a polymer MRR by using sNIL with a PDMS soft mold [64]. They fabricated SU-8 patterns on the Si substrate by using EBL followed by polymer replication of the PDMS soft mold. They fabricated the PS MRR on the OG-125 (Epotek, n ~ 1.456) coated Si substrate by sNIL using the PDMS soft mold pressing with a force of 25 N for 20 min. They also fabricated the SU-8 MRR by using the same sNIL method with UV curing to polymerize the SU-8 structure. The MRRs have a dimeter of 400 μm, a width of 1.9 μm, a height of 1.5 μm, and a gap of 350 nm. Their Q-factors are 1.1 × 10^4^ and 6.5 × 10^3^ at 1.55 μm for the PS MRR and the SU-8 MRR, respectively. Teng et al. also reported a polysiloxane-liquid (PSQ-L) MRR on the Si substrate with the Q-factor of 4.2 × 10^4^ at 1.55 μm by using sNIL with the PDMS soft mold [65]. They fabricated a lower cladding layer with a low refractive index PSQ-L by sNIL and then spun-coated a core layer of a high refractive index PSQ-L. While PDMS emerged as a typical choice for the material of the soft mold in the sNIL process, PFPE materials can be an alternative to the PDMS thanks to its lower surface energy and ideal elastic modulus [69]. sNIL with PFPE soft molds demonstrated polymer MRRs with the Q-factor of 2.0 × 10^4^ and 2.3 × 10^4^ at 890 nm using a commercial polymer OrmoCore (*Microresist Technology*) [66, 68]. In addition, roll-to-roll sNIL using the PFPE mold enabled low-cost and high throughput fabrication of UV-curable Norland Optical Adhesives (NOAs) MRR with the Q-factor of 5.8 × 10^4^ at 1.5 μm [67].

Recently, PS MRRs with high Q-factor up to 1.4 × 10^5^ at 780 nm were fabricated by sNIL using a PDMS soft mold and implemented as the ultrasound detector with high sensitivity and broad frequency bandwidth for PAM and PACT [31, 35]. Li et al. successfully demonstrated longitudinal cortical PAM imaging of mouse brain by applying a protection cladding layer of PDMS on the PS MRR fabricated on the transparent quartz substrate [31]. The MRR pattern with the gap distance of 100 nm and a high aspect ratio of 8:1 was well transferred from the master Si mold to the PS layer on the thin quartz substrate by using sNIL with the PDMS soft mold (Fig. 8b–g). Their MRR ultrasound detector achieved the axial resolution of 3.57 μm and the NEP of 0.49 Pa in the frequency bandwidth of 166 MHz.

**Fig. 9:**
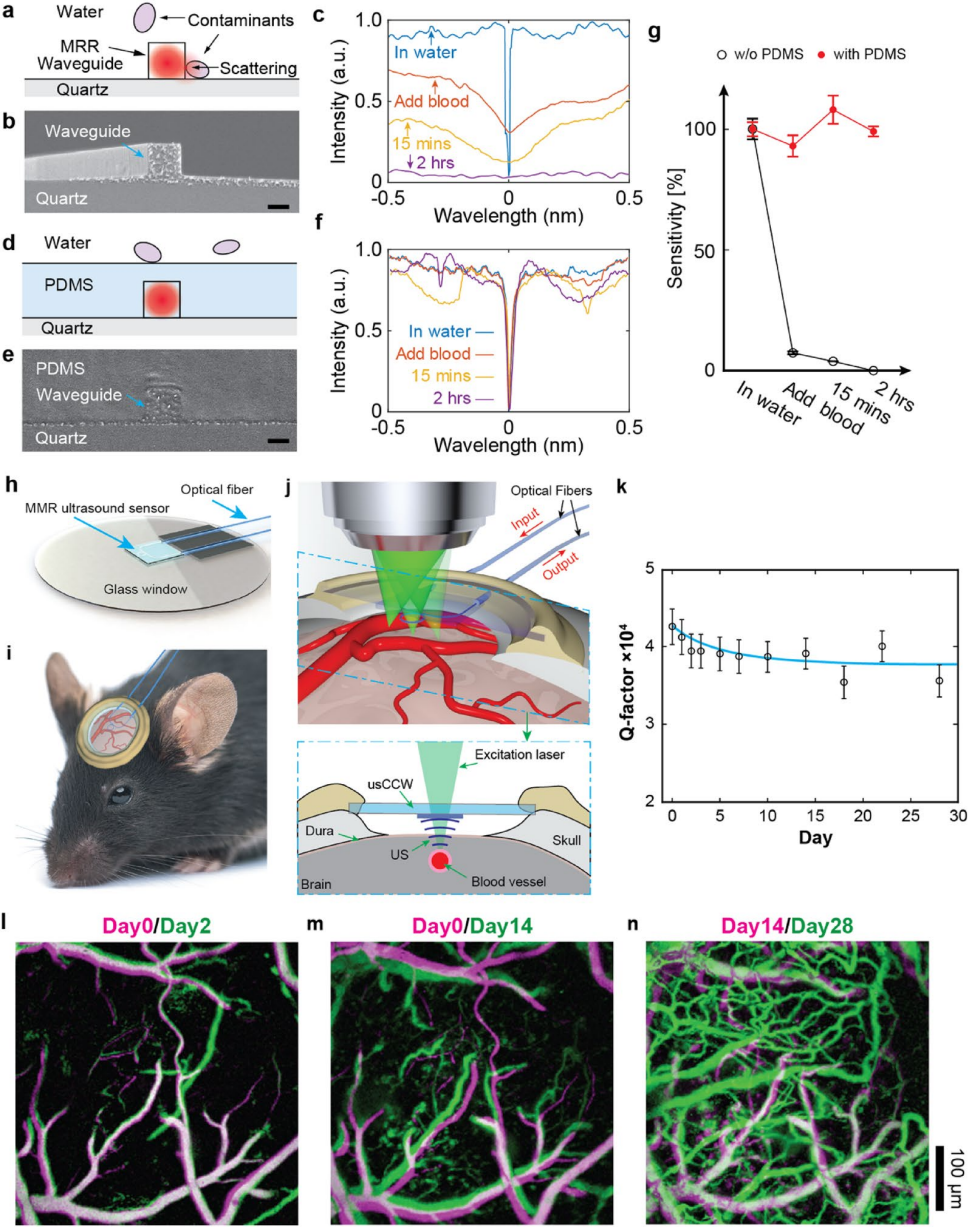
**a** A schematic illustration of potential contaminants attached to the waveguide. **b** A cross-sectional SEM image of the PS MRR in air. Scale bar 500 nm. **c** Optical resonance spectrum when unprotected MRR is exposed to whole blood for 2 h. **d** A schematic illustration of the MRR with the PDMS protection layer. **e** A cross-sectional SEM image of the PS MRR with the PDMS protection layer. Scale bar 500 nm. **f** Optical resonance spectrum when the MRR with the protection layer is exposed to whole blood for 2 h. **g** The quantitative comparison of the detection sensitivity in whole blood. **h** A schematic of the packaged MRR on the glass coverslip with the optical fibers. **i** The polymer MRR ultrasound detector for chronic cranial window mounted on the mouse’s forehead. **j** Illustration of optical scanning through the transparent polymer MRR. **k** Marginal reduction in the Q-factor of the MRR mounted on the mouse’s forehead for the 28-day-period. **l–n** Longitudinal PAM images of cortical vasculature of the mouse brain (Reproduced with permission from [34])

**Fig. 10:**
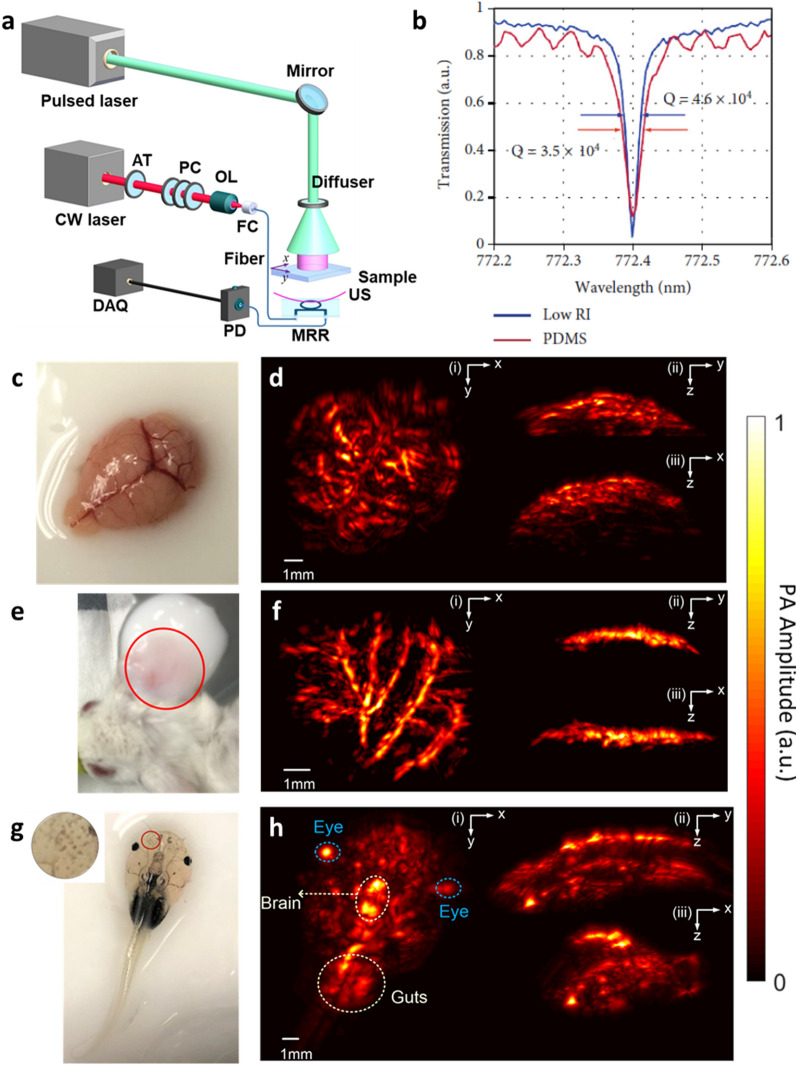
**a** A schematic illustration of 3D PACT system with the polymer MRR ultrasound detector. CW: continuous wave; AT: attenuator; OL: objective lens; PC: polarization controller; FC: fiber coupler; DAQ: data acquisition; APD: avalanche photodetector; US: ultrasound; SP: scanning plane. **b** The resonance spectrum and the Q-factor of the MRR with the PDMS cladding and the low refractive index polymer cladding. **c** Photograph of the perfused mouse brain with different image planes. **d** MAP PA images of the perfused brain. **e** Photograph of the mouse ear. **f** MAP PA images of the mouse ear. **g** Photograph of Xenopus Laevis tadpole. Inset is a close-up image of the skin pigments. **h** MAP PA images of the tadpole (Reproduced with permission from [35])

When the polymer MRR ultrasound detector is exposed to a biological environment, contaminants from biological tissues can adhere to the surface and the gap of the MRR. This results reduction of the Q-factor and the loss of the resonance spectrum due to the scattering loss and compromised coupling between the ring and the bus waveguide (Fig. 9a–c). They added the PDMS as a protective layer to prevent contaminations of the polymer MRR in biological environment such as blood (Fig. 9d, e). The protection layer enables the Q-factor and the ultrasound detection sensitivity maintained in the blood for 2 h (Fig. 9f, g). Their distinct optical transparency, miniaturized form-factor, and the protection layer allow the polymer MRR ultrasound detector to apply for longitudinal PA imaging and in vivo study. They implanted the packaged polymer MRR ultrasound detector based chronic cranial window on a mouse’s forehead (Fig. 9h, i). The polymer MRR was packaged on the glass substrate with a SMF (S630-HP, *Thorlabs*) as the input port and a MMF (GIF625, *Thorlabs*). The MRR ultrasound detector fitted well inside the narrow space between the dura and glass window and the excitation laser for PA generation can illuminate and scan across the region of interest on the cortex thanks to its miniaturized form-factor and optical transparency (Fig. 9j). The Q-factor of the MRR shows marginal reduction from 4.2 × 10^4^ to 3.6 × 10^4^ over the 28-day-period, which confirms the stability of the ultrasound detector for in vivo applications (Fig. 9k). They successfully conducted long-term intravital PAM imaging of live mouse brains for 28 days using the polymer MRR ultrasound detector as the chronic cranial window (Fig. 9l–n). In addition, Rong and Lee et al. demonstrated in vivo deep-tissue high-frequency 3D PACT imaging using the PS MRR with the protection layer as a point-like ultrasound detector thanks to its miniaturized form-factor and high sensitivity [35]. Figure 10a illustrates the PACT system with the MRR ultrasound detector. A nanosecond pulsed laser (Q-smart 850, *Quantel*) was used for PA excitation laser. The beam of the pulsed laser was expanded and homogenized to an expanded diameter of 1 cm over a sample by using an optical diffuser (DG10-220-MD, *Thorlabs*). The sample was mounted on a three-axis motorized scanning stage (L-509, *PI*) that can provide a scanning range over a FOV of 26 by 26 mm^2^. The MRR ultrasound detector was placed 4 mm beneath the sample with water in between as acoustic coupling medium. They achieved the lateral resolution of 114 μm and the axial resolution of 57 μm in the frequency bandwidth of 23 MHz using the PS MRR with the Q-factor of 4.6 × 10^4^. The refractive index of the protection layer affects the Q-factor of the polymer MRR when the other conditions are the same because higher refractive index contrast between the core material and the cladding material reduces the scattering loss through the sidewall of the ring waveguide. They further improved the Q-factor of the MRR from 3.5 × 10^4^ to 4.6 × 10^4^ by changing the protection material from PDMS to the low refractive index polymer (MY-131-MC, *MY Polymer*) (Fig. 10b). They also demonstrated 3D PACT imaging for ex vivo mouse brain and in vivo mouse ear and *Xenopus Laevis* tadpole (Fig. 10c–h). 3D PA images clearly showed major vasculature structures in the mouse brain and ear and major blood vessels, eyes, brain, guts, and skin pigments.

## Light coupling methods

In the configuration of the MRR based ultrasound detector, the excitation laser for driving the MRR is coupled into the input port of the bus waveguide and the transmitted light signal from the output port of the bus waveguide is collected by the photodiode detector. Optimal coupling method can achieve high coupling efficiency for PA signal improvement and facilitate packaging of MRRs for practical PA imaging applications. In order to couple the light and collect the transmitted signal, free space coupling with objective lenses [49], coupling with tapered optical fibers [30, 32, 37], and butt-coupling of optical fibers [33, 58, 70] were used for MRR based ultrasound detection and PA imaging. The free space coupling method with objective lenses can provide high in- and out- coupling efficiency, but this method is limited to use for practical PA imaging systems due to its difficulty to package together with the MRR ultrasound detector. The coupling with tapered optical fibers enables high coupling efficiency by closely matching the mode profile and facilitates packaging with the MRR in PA imaging applications. However, it requires dedicated equipment to fabricate tapered optical fibers with desired tip diameters. Although the butt-coupling of optical fibers has lower coupling efficiency compared to other methods due to mismatch of the mode profiles between the SMF and the bus waveguide of the MRR, this method has been widely used in recent PA imaging applications [31, 35] thanks to its simplicity and cost effectiveness. The coupling efficiency in the butt-coupling can be further improved by using one-dimensional tapered bus waveguides for mode conversion to reduce the mode mismatch between the SMF and the bus waveguide of the MRR.

## Conclusions

In conclusion, it took more than 20 years to transform the polymer MRR ultrasound detector from a primitive concept into a viable high-performance device. Design innovations and improvements to the nano-fabrication process not only substantially improved its performance, but also significantly reduced the overall manufacturing cost, making it more accessible to the broader research community. Polymer MRR based optical ultrasound detectors have demonstrated unique advantages in high sensitivity, broad frequency bandwidth, smaller detector size, and optical transparency. More recently, we have witnessed a steady increase in the adaptations of the polymer MRR ultrasound detectors by many research groups, in developing novel PA imaging applications. For the interest of the readers, we have summarized the characteristic performance of the polymer MRR ultrasound detectors being reviewed and the associated nano-fabrication process in Table 1. We hope this review can be useful for researchers who wish to learn more about the polymer MRR based optical ultrasound detectors and use them for their PA imaging applications.

**Table 1:** Summary of nanofabrication methods of polymer MRRs for optical ultrasound detectors and their key parameters

Fabrication method	Substrate	Core material	Detector diameter	Waveguide dimension (width × height)	Q-factor	Working wavelength	Frequency bandwidth	NEP	PA imaging resolution	Application	Year [Refs]
NIL SiO_2_ on Si mold	SiO_2_ on Si	PS	60 μm (racetrack)	2.3 μm x 1.8 μm	1.0 × 10^3^	1.55 μm	10 MHz	N/A	N/A	US	2004 [49]
	SiO_2_ on Si	PS	95 μm	2.4 μm x 1.85 μm	6.0 × 10^2^	1.55 μm	40 MHz	150 kPa	N/A	US	2007 [37]
	SiO_2_ on Si	PS	100 μm	2 μm x 2 μm	6.0 × 10^3^	1.55 μm	90 MHz	230 Pa	N/A	US	2008 [50]
	SiO_2_ on Si	PS	100 μm	2 μm x 2 μm	5.0 × 10^3^	1.55 μm	70 MHz	4.1 kPa	Lateral: 150 μm Axial: 90 μm	PAT	2008 [51]
	SiO_2_ on Si	PS	100 μm	2 μm x 2 μm	6.0 × 10^3^	1.55 μm	16 MHz	111.4 Pa	Lateral: 209 μm Axial: 81 μm	PAT	2009 [52]
	SiO_2_ on Si	PS	100 μm	2 μm x 2 μm	6.0 × 10^3^	1.55 μm	74 MHz	200 Pa	Lateral: 146 μm Axial:50 μm	PAT	2011 [57]
	Quartz	PS	100 μm	2 μm x 2 μm	5.0 × 10^3^	1.55 μm	51 MHz	N/A	Transverse:750 μm Radial: 21 μm	PA endoscopy	2011 [53]
NIL Si mold	SiO_2_ on Si	PS	90 μm	N/A	1.5 × 10^5^	1.55 μm	74 MHz	88 Pa	N/A	US	2011 [28]
	SiO_2_ on Si	PS	60 μm 40 μm	1 μm x 1.4 μm	4.0 × 10^5^ 7.0 × 10^4^	780 nm	74 MHz	21.4 Pa 100 Pa	Axial: 75 μm Axial: 50 μm	PAT	2011 [29]
	SiO_2_ on Si	PS	40 μm	1 μm x 1.4 μm	7.0 × 10^4^	780 nm	74 MHz	100 Pa	Lateral: 55 μm Axial:50 μm	PAT	2011 [57]
	SiO_2_ on Si	PS	60 μm	1 μm x 1.4 μm	3.0 × 10^5^	780 nm	74 MHz	29 Pa	Lateral: 5 μm Axial: 8 μm	PAM	2011 [58]
	SiO_2_ on Si	PS	60 μm	1 μm x 1.4 μm	3.0 × 10^5^	780 nm	74 MHz	29 Pa	Lateral: 17.5 μm Axial: 20 μm	PAM	2012 [59]
	SiO_2_ on Si	PS	95 μm	2.4 μm x 1.85 μm	N/A	1.55 μm	28 MHz	N/A	Lateral: 305 μm Axial: 169 μm	PA-US	2012 [61]
	SiO_2_ on Si	PS	60 μm	1 μm x 1.4 μm	1.3 × 10^5^	780 nm	350 MHz	105 Pa	Lateral: N/A Axial: 2.7 μm	PAM	2014 [60]
EBL	Quartz	SU-8	60 μm	0.8 μm x 0.8 μm	1.0 × 10^4^	780 nm	140 MHz	6.8 Pa	Lateral: N/A Axial: 5.3 μm	PAM	2014 [30]
	Quartz	SU-8	60 μm	0.8 μm x 0.8 μm	4.8 × 10^3^	780 nm	250 MHz	352 Pa	Tangential: 15.7 μm radial: 4.5 μm Axial: 16 μm	PA endoscopy	2014 [33]
	Quartz	SU-8	60 μm	0.8 μm x 0.8 μm	1.0 × 10^4^	780 nm	280 MHz	6.8 Pa	Lateral: 0.73 μm Axial: 2.12 μm	Multimodal PAM	2015 [32]
MPL	Quartz	IP-Dip	60 μm	4 μm (diameter)	3.4 × 10^3^	1.55 μm	10 MHz	N/A	N/A	US	2017 [46]
sNIL PDMS mold	Quartz	PS	80 μm	0.8 μm x 0.8 μm	1.4 × 10^5^	780 nm	166 MHz	0.49 Pa	Axial: 3.57 μm	PAM	2019 [34]
	Quartz	PS	80 μm	0.8 μm x 0.8 μm	4.6 × 10^4^	780 nm	23 MHz	81 Pa	Lateral: 114 μm Axial :57 μm	PACT	2022 [35]

N/A: not available. US: ultrasound detection

## Data Availability

Not applicable.
